# Engineered MED12 mutations drive uterine fibroid-like transcriptional and metabolic programs by altering the 3D genome compartmentalization

**DOI:** 10.21203/rs.3.rs-2537075/v1

**Published:** 2023-02-09

**Authors:** Kadir Buyukcelebi, Xintong Chen, Fatih Abdula, Alexander Duval, Harun Ozturk, Fidan Seker-Polat, Qiushi Jin, Ping Yin, Yue Feng, Jian-Jun Wei, Serdar Bulun, Feng Yue, Mazhar Adli

**Affiliations:** Northwestern University; Northwestern University; Northwestern University; Northwestern University; Northwestern University; Northwestern University; Northwestern University; Northwestern University; Northwestern University; Northwestern University; Northwestern University; Northwestern University; Northwestern University

## Abstract

Uterine fibroid (UF) tumors originate from a mutated smooth muscle cell (SMC). Nearly 70% of these tumors are driven by hotspot recurrent somatic mutations in the *MED12* gene; however, there are no tractable genetic models to study the biology of UF tumors because, under culture conditions, the non-mutant fibroblasts outgrow the mutant SMC cells, resulting in the conversion of the population to WT phenotype. The lack of faithful cellular models hampered our ability to delineate the molecular pathways downstream of *MED12* mutations and identify therapeutics that may selectively target the mutant cells. To overcome this challenge, we employed CRISPR knock-in with a sensitive PCR-based screening strategy to precisely engineer cells with mutant *MED12* Gly44, which constitutes 50% of *MED12* exon two mutations. Critically, the engineered myometrial SMC cells recapitulate several UF-like cellular, transcriptional and metabolic alterations, including enhanced proliferation rates in 3D spheres and altered Tryptophan/kynurenine metabolism. Our transcriptomic analysis supported by DNA synthesis tracking reveals that *MED12* mutant cells, like UF tumors, have heightened expression of DNA repair genes but reduced DNA synthesis rates. Consequently, these cells accumulate significantly higher rates of DNA damage and are selectively more sensitive to common DNA-damaging chemotherapy, indicating mutation-specific and therapeutically relevant vulnerabilities. Our high-resolution 3D chromatin interaction analysis demonstrates that the engineered *MED12* mutations drive aberrant genomic activity due to a genome-wide chromatin compartmentalization switch. These findings indicate that the engineered cellular model faithfully models key features of UF tumors and provides a novel platform for the broader scientific community to characterize genomics of recurrent *MED12* mutations and discover potential therapeutic targets.

## Introduction

Uterine fibroids (UF), also called leiomyomas, are benign monoclonal neoplasms of the myometrium that represent the most common gynecologic tumors among reproductive-age women^1,2^ . By the age of 50, more than 70% of all women (70% white and > 80% black) develop at least one fibroid tumor. UF are non-malignant and not symptomatic in the majority of cases; however, in 15–30% of cases, they disrupt normal uterine functions, resulting in a wide range of severe health problems, including excessive uterine bleeding, anemia, defective implantation of an embryo, recurrent pregnancy loss, preterm labor and obstruction of labor and may mimic or mask malignant tumors in 15 to 30% of reproductive-age women^2^. Few medical treatments are available for UF, and many women opt to undergo a surgical hysterectomy. However, such procedures create significant emotional stress on individual patients and a substantial financial burden on society. These practices are estimated to cost $5.9 -$34.4 billion in the USA alone^3^.

At the genomic level, UF tumors have a relatively low mutational burden with few recurrent genetic mutations. Significantly, nearly 70% of UF tumors harbor somatic mutations in the *MED12* gene, encoding the Mediator Complex Subunit 12 (*MED12*)^4^. Furthermore, translocation in the high mobility group AT-hook 2 (*HMGA2*) gene, recurrent loss of fumarate hydratase (*FH*), and deletion of collagen *COL4A5*-*COL4A6* gene are among other recurrent somatic genetic alterations^5^. Integrated data analysis reveals mutation-specific distinct driver gene expression programs and biomarkers in UF^6^, indicating the importance of studying and revealing the mutation-specific aberrant driver targets in UF.

The mediator complex regulates transcription initiation and elongation by bridging the regulatory elements (enhancers) with gene promoters and facilitating the RNA polymerase II transcriptional initiation^7,8^. Therefore, the complex is critical for conveying the information between gene-specific transcription factors and the basal RNA polymerase II transcriptional assembly^7,8^. The mediator is structurally assembled from a set of 26 core subunits in humans arranged into three distinct modules: head, middle, and tail, that bind to Pol II as a “holoenzyme”^9–12^. The proper function of these core modules is regulated by a detachable CDK8 kinase module^8,12^, which consists of MED12, CDK8, Cyclin C, and MED13. MED12-dependent CDK8 activation is a critical regulator of the Mediator complex, as oncogenic MED12 mutations disrupt its allosteric regulation of CDK8^12,13^. MED12 connects Cyclin C-CDK8 to the core Mediator and is required for the kinase activity of the CDK8 submodule^7,14^. Recent findings indicate that the MED12-containing CDK8 subcomplex can also function independently of the mediator complex^14^. MED12 is involved in several cancer-related signaling pathways related to various nuclear receptors, Wnt, and Sonic Hedgehog^15–17^.

MED12 consists of 45 exons and is located in the *q13* arm of the X chromosome. Initial exome sequencing revealed that 70% of UF patients display recurrent MED12 mutations^4^, making it the most frequently altered gene UF^4,5,18^. This finding has been replicated in several studies of various ethnic groups, reporting MED12 mutation frequencies in up to 92% of all UF^18^. Significantly, UF-associated MED12 mutations are missense mutations that affect the highly conserved region in exon two. The majority of UF-linked MED12 hotspot mutations (more than 60% of all MED12 mutations) occur at Gly-44 (> 50%) and two other residues (Leu-36 and Gln-43)^4,18^. The recurrent alteration in patient samples and the data from the engineered mouse model strongly suggest that altering the Glycine at the 44 amino acid leads to a gain of function “oncogene” mutation that drives UF tumorigenesis^19^.

UF tumors are believed to originate mono-clonally from a single mutated smooth muscle cell (SMC). However, the mutated SMC co-exists with non-mutated tumor-associated fibroblasts (TAF) *in vivo*^20^. Critically, when cultured *in vitro*, these WT fibroblasts outgrow the mutant cells, resulting in the loss of mutant cells and conversion of the population to WT phenotype^21,22^. This hampered the development of cellular models to deeply characterize MED12 mutations, understand the molecular pathways downstream of these mutations and develop therapeutic targets to inhibit UF tumorigenesis. To overcome this formidable challenge, we used CRISPR-mediated homology-directed repair (HDR) to precisely engineer a G44 mutation (Gly ◊ Asn) in a disease-relevant myometrial SMC line. Critically, the cellular, molecular and metabolic profiling of the engineered cells highlights that MED12 G44 mutation recapitulates major cellular and molecular phenotypes of MED12 mutant UF tumors, including altered proliferation, fibroid-like transcriptional program, and genomic instability, indicating that the engineered cells faithfully model several molecular features of UF biology. Significantly, our transcriptional and 3D genome mapping and analysis show that MED12 Gly4 mutations lead to genome-wide 3D chromatin organization and genome compartmentalization.

## Results

### Engineering MED12 Gly44 mutation in clonal myometrium smooth muscle cells:

**Figure 1:**
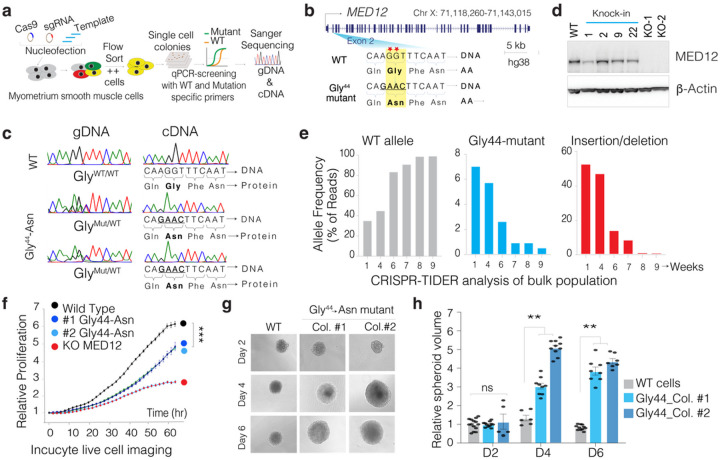
CRISPR-engineered recurrent MED12 Gly44 mutation results in aberrant cell proliferation. **a.** Schematics shows the strategy used in this study to CRISPR engineer a UF-relevant specific MED12 mutation in an immortalized smooth muscle cell. **b.** Schematics of the MED12 gene and most frequently observed mutations (red asterisk) are shown at the exon two region. The WT and the homology-directed repair (HDR) DNA template are shown for the intended MED12 → Gly44 Asn mutation. **c.** Chromatograms show the sanger sequencing results from genomic DNA and cDNA from WT cells and multiple CRISPR-engineered MED12 Gly 44 clones. **d.** Western blot showing *MED12* and b-actin protein levels in control, CRISPR-engineered cells. **e.** CRISPR-TIDE analysis shows the frequency of DNA sequencing reads with WT sequence, homology-directed repaired (*MED12* Gly44 mutant), and indels assessed over several weeks from a population of cells. **f.** InCusyte live cell imaging results show relative proliferation rates of control WT and *MED12* Gly44 mutant cells in 2D culture conditions. **g.** Representative images of 3D spheres of WT and *MED12* Gly44 mutant cells in 3D culture conditions. **h.** The bar plot shows relative spheroid volumes from WT and *MED12* Gly44 mutant cells (**p<0.01,***p<0.001, Two-sided unpaired t-test)

Notably, more than 50% of all MED12 exon two mutations are in the 44th codon and mutate Glycine into at least six other aa, indicating the significance of Glycine at this position for proper MED12 function. We set out to use CRISPR to knock in a respective mutation containing a DNA template in exon 2 of the MED12 gene in immortalized myometrial SMC line that retains the expression of various markers of primary SMC^23^. We utilized a CRISPR-based knock-in and qPCR-based single-cell colony selection strategy^24^ (Fig. 1a, **Supporting** Fig. 1) to introduce Gly-44 ◊ Asn mutation in exon 2 of MED12 and select single-cell colonies. The knock-in template is designed to also disrupt the PAM sequence while altering the aa at the 44th codon. We transiently delivered WT Cas9, sgRNA, and a custom-designed knock-in single-stranded DNA (ssDNA) oligo template through nucleofection. We then single-cell-sorted, grew individual nucleofected cells and performed qPCR-based colony screening on genomic DNA from > 400 single-cell colonies (**Supplementary Fig. 2**), we obtained multiple clonal cell lines that are homozygous and heterozygous for Gly-44 mutations (Fig. 1b–c). We validated the WT and mutant allele frequency using CRISPR-TIDER analysis (**Supplementary Fig. 3**). Critically, since *MED12* is on the X chromosome, one allele undergoes random epigenetic inactivation. Thus, we performed additional screening from the cDNA of the single-cell clones to identify several clonal cell lines expressing the mutant Gly-44 MED12 in the mRNA as validated by cDNA sequencing (Fig. 1c). Western blot analysis shows that the Gly44 mutation does not alter MED12 protein stability (Fig. 1d).

#### MED12 Gly44 mutation recapitulates UF-specific proliferation defects.

A formidable challenge in establishing an *in vitro* model of fibroid tumors has been the rapid disappearance of cells carrying *MED12* mutations when the fibroid tumor cells are cultured *in vitro*^21,22^. This is believed to be due to a reduced proliferative capacity in 2D culture conditions *in vitro*. To test whether such a slower proliferation defect can cause the disappearance of mutant cells from the population, we initially analyzed the mixed population of cells right after the CRISPR knock-in. Our targeted sequencing and CRISPR-TIDER analysis indicated that the mixed population contained ~ 36% WT allele, 7% knock-in allele (Gly 44 mutant), and 57% indel alleles (likely MED12 KO) after initial CRISPR editing. We then continuously cultured this mixed population and performed targeted sequencing at exon 2 of MED12 at the 4th, 6th, 7^th,^ and 9th weeks after the initial gene editing. Significantly, we observed that the mixed population reached ~ 100% WT allele while gradually losing both the G44 mutant and the indel alleles (Fig. 1e), supporting the observed phenotype of MED12 mutant primary SMC cells^21,22^. To quantify the proliferation defects in these cells more precisely, we studied clonally expanded pure populations (from single cells) of MED12 WT, Gly44 mutant, or KO SMC cells. We used Incucyte live cell imaging platform to robustly detect cell proliferation defects by monitoring individual nuclei counts over several days. Critically, the Gly44 mutant cells are less proliferative than WT cells but better than the KO cells (Fig. 1f). The time course experiment indicates that the WT cells double in ~ 23 hrs, whereas the MED12 Gly44-mutant cells double every ~ 32 hrs in 2D adherent conditions. Although interesting, these findings raised the question of how such a mutation drives UF tumorigenesis if it leads to reduced cell proliferation. We, therefore, tested whether the reduced proliferation is due to the restrictive 2D culture conditions.

#### The MED12 Gly44 mutation increased cell-autonomous and non-autonomous proliferation capacity.

MED12 Gly 44 mutations are causal in inducing higher cell proliferation and, eventually, tumor formation in humans and mice. To understand whether the reduced proliferation phenotype is an artifact of 2D culture conditions, we cultured the WT and the engineered mutant cells in 3D spheroid conditions. Notably, the MED12 mutants formed significantly larger spheroids than WT cells (Fig. 1g–h), indicating the significance of culture conditions in studying MED12 mutations. On average, we observed that MED12 mutant spheres grew 2–4 times larger than the WT colonies. At the same time, the KO cells did form any spheres (not shown), indicating that the engineered MED12 Gly44 mutation is UF-relevant and is a gain-of-function mutation.

Notably, the MED12 mutant UF is composed of mutant SMC and non-mutant tumor-associated fibroblasts and stromal cells at nearly equal rates^20^, suggesting that the mutant cells cause the proliferation of non-mutant cells in a cell non-autonomous fashion. To test whether the engineered mutant cells will also increase the proliferation in non-mutant cells, we performed co-cultured experiments with fluorescently labeled (mCherry) non-mutant cells. We quantified the rate of proliferation in fluorescently labeled non-mutant cells. In line with the data in Fig. 1g, we observed larger volume spheroids when mutant cells were cultured with non-mutant cells. We dissociated the spheroids to reveal whether the larger spheroid formation is partly due to the increased proliferation of non-mutant cells. We counted the number of mCherry(+) cells from each condition. This analysis indicated that mutant cells enhance the proliferation capacity of the non-mutant cells in the microenvironment (**Supplementary Fig. 4**).

#### The engineered MED12 Gly44 mutation alters the global metabolism in smooth muscle cells.

**Figure 2:**
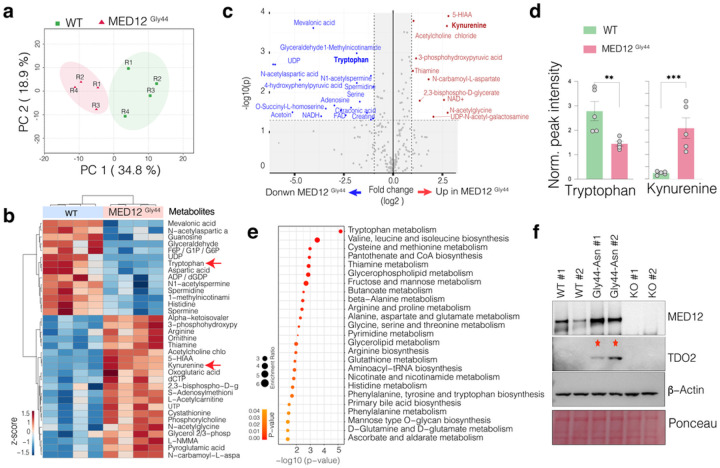
MED12 Gly44 mutations result in fibroid-relevant metabolic reprogramming. **a.** Dimension reduction shows the top two principal components that capture 34.8 % and 18.9 % variability in the global metabolic differences in the indicated samples. **b.** The heatmap shows the top enriched and depleted metabolites in indicated cells. **c.** The scatterplot shows statistical significance (−log10 p-value) versus the magnitude of change (−log 2-fold change) in metabolites of *MED12* Gly 44 cells vs. WT cells. **d.** The bar plots show the normalized intensity of LC-MS peaks for Tryptophan and Kynurenine metabolites in indicated cells. **e.** Dot plots show the p-value and enrichment levels of top metabolic terms for the differentially regulated metabolites between WT and *MED12* mutant cells. **f.** Western blots show *MED12* and TDO2 protein levels. B-actin and Ponceau S staining is shown for loading control..(**p<0.01,***p<0.001, Two-sided unpaired t-test)

The altered proliferation rates are driven by overall cellular metabolic and transcriptional reprogramming. Uterine fibroid tumors have distinct metabolic and bioenergetic needs. For example, uterine fibroid tumors are known to be depleted of Tryptophan but replete with Kynurenine, a product of Tryptophan metabolism^25^. We, therefore, set out to investigate global metabolite levels in WT and mutant cells by performing Liquid Chromatography and Mass Spectrometry (LC-MS) to acquire signals from a broad spectrum of water-soluble metabolites (250 plus targets). The principal component analysis (PCA) of overall metabolite contents (**Supplementary Table 1**) indicates that the WT and MED12 mutant cells have distinct metabolic states as each formed distinct clusters based on the top two principal components (Fig. 2a), indicating the significant metabolic differences between these two cells. Specifically, MED12 mutation leads to the downregulation of 14 metabolites while upregulating, a larger number, of 21 metabolites (Fig. 2b–c).

Critically, increased 5-HIAA, Kynurenine, thiamine, N-carbomyl-L-aspartate, and reduced Tryptophan, mevalonic acid, N-acetylaspartic acid, glyceraldehyde were the top differentially regulated metabolites. Tryptophan is an essential amino acid that the body needs to acquire from the diet and is metabolized into Kynurenine. Our detailed quantifications show that while Tryptophan is significantly (p < 0.01, t-test) depleted in MED12 mutant cells, Kynurenine levels are abnormally elevated (p < 0.01, t-test) (Fig. 2d). In line with this, Tryptophan metabolism was the top enriched metabolic term when all differentially regulated metabolites were analyzed together (Fig. 2e). Notably, our group previously reported that the reduced Tryptophan levels in MED12 mutant uterine fibroids are driven by increased levels and activity of an enzyme called *Tryptophan 2,3-Dioxygenase-2 (TDO2)* that converts Tryptophan into Kynurenine^26^. We, therefore, tested whether our engineered cells have elevated levels of TDO2 protein. Critically, the western blot analysis shows that the mutant cells have markedly increased levels of TDO2, whose levels are not detectable in WT and the MED12 KO cells (Fig. 2f). These findings highlight that the engineered Gly44 mutation recapitulates known metabolic reprogramming in primary UF tumors^25,26^.

#### The MED12 Gly44 mutant cells have distinct transcriptional states.

**Figure 3:**
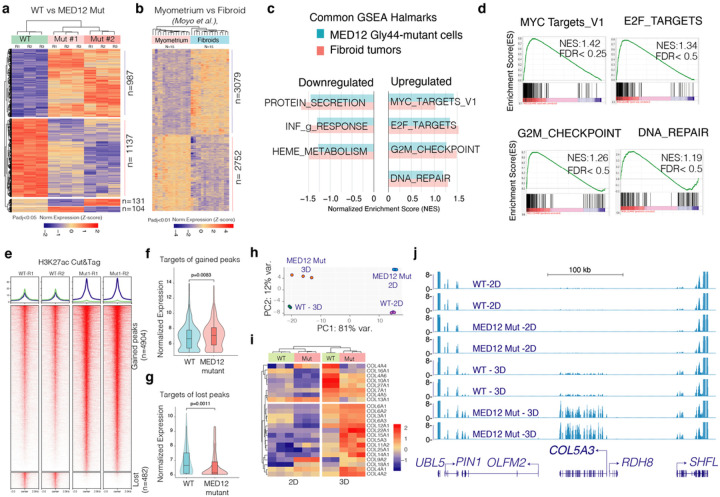
MED12 Gly44 mutations cause a fibroid-like transcriptional program that is further enhanced in 3D conditions. **a-b.** Heatmaps show differentially expressed genes in engineered MED12 mutant cells (a) and primary fibroid tumors. c. The horizontal bar plot shows common hallmarks from Gene Set Enrichment Analysis (GSEA) in engineered MED12 mutant cells and fibroid tumors. **d.** GSEA plots show enriched hallmarks in MED12 mutant vs. WT cells. **e.** Heatmaps show regulatory genomic regions differentially marked by the H3K27ac mark (Cut & Tag signal intensity). **f-g.** Violin plots and box plots show expression levels of gene targets (<10 kb away from the peaks) of gained (f) and lost peaks (g) in MED12 mutant cells. **h.** The dot plot shows the top two principal components that explain the largest variation in gene expression programs of indicated cell types under two different culture conditions. **i.** The heatmap shows differentially regulated collagen genes in WT and MED12 mutant cells in 2D and 3D conditions. **j.** The RNA-Seq tracks show the gene expression level of COL5A3 and neighboring genes in WT and MED12 mutant cells under 2D and 3D conditions.

We next assessed overall transcriptional reprogramming downstream of MED12 Gly44 mutation by performing RNA-Seq in two separate MED12 Gly44-mutant clones, the WT clones and the MED12 KO cells in triplicate. Notably, > 90% of the genes (2124/2359) between WT and mutant cells were consistent across the mutant clones, indicating the robustness of MED12 mutation-driven transcriptional changes. Less than 5% (104 or 131/2359) of differentially expressed genes were clone-specific, indicating the robustness of MED12 mutations and minimal clonal heterogeneity compared to WT clones. Differential expression analysis showed that MED12 mutation drives the differential expression of ~ 2000 genes, consistent in both MED12 mutant clones (987 upregulated and 1137 downregulated, p-adj < 0.05) (Fig. 3a). Most critically, the MED12 Gly44 mutations resulted in differential expression of a distinct set of genes compared to MED12 knock-out cells (**Supplementary Fig. 5**), supporting the overall hypothesis that the UF-associated MED12 mutations are not loss of function, but a gain of function mutations.

#### The aberrant transcriptional program of MED12 Gly44 mutant cells is reminiscent of fibroid tumors.

To assess whether these differentially expressed genes are comparable and relevant to fibroid tumors, we analyzed them with the recent gene expression program of normal myometrium (n = 15) and *MED12* mutation harboring fibroid tumors (n = 15) reported by *Moyo et al*. We detected ~ 5500 differentially expressed genes (P-adj < 0.01) between normal myometrium and UF samples (Fig. 3b). Notably, the gene set enrichment analysis (GSEA) highlighted that several hallmarks are shared among the mutant cells and primary fibroid tumors. For example, hallmarks of cell cycle-related genes, MYC and E2F target genes, and DNA repair genes are substantially upregulated in the mutant cells as well as primary UFs. On the other hand, protein secretion and heme metabolism genes are among the most downregulated genes. (Fig. 3c). In line with GSEA, the gene ontology (GO) analyses on differentially expressed genes in Gly44 mutant cells and fibroid tumors identified a common set of biological processes between these two samples. For example, the upregulated genes in Gly44-mutant and fibroid tumors are enriched for cell cycle and DNA replication-related gene ontology (GO) terms (**Supplementary Fig. 6**). Conversely, the downregulated genes in our MED12 mutant cells and primary fibroids included a group of cell adhesion and extracellular matrix (ECM) reorganization genes, indicating that, Gly44 mutation also recapitulates the abnormal ECM feature of human UFs^27,28^.

We next assessed the epigenome of these cells to see whether MED12 mutation leads to aberrant gene expression changes through the altered epigenome. Since MED12 is a critical regulator that mediates promoter-enhancer interaction, we acquired a genome-wide map of Histone H3 Lysine 27 (H3K27ac) histone modification which marks active enhancers and promoters^29,30^ by Cut & Tag^31^. Notably, differential peak analysis identified that 4904 peaks gained the H3K27ac mark, while 482 of the regulatory elements nearly lost all H3K27ac signal (Fig. 3e), indicating that MED12 Gly44 mutation results in increased genomic activity at most regulatory elements. To identify whether this change in H2K27ac chromatin state had a corresponding change in target gene expression, we analyzed the expression change in their target genes mapped by their genomic proximity to the H3K27ac peak (< 10kb). Notably, the targets of gained peaks in MED12 mutant cells had a significantly higher expression in these cells compared to WT cells (Fig. 3f). Conversely, the gene targets of lost peaks in MED12 mutants dramatically reduced their expression in these cells (Fig. 3g). These findings indicated that MED12 mutations induced gene expression changes is, in part, due to reprogrammed epigenome, at least of the H3K27ac chromatin states.

#### MED12 mutations lead to enhanced expression of collagen genes in 3D culture conditions.

Fibroid tumors are known to have aberrant remodeling of extracellular matrix and significantly higher production of collagen^20,27,32^. Recent transcriptome and epigenome profiling by Moyo et al.^33^, also highlighted significantly higher expression of collagen genes in fibroid tumors. Surprisingly, we did not find higher expression of collagen genes in our 2D cultured cells. We, therefore, wondered whether this is due to culture conditions. We thus obtained RNA-seq expression profiles of these cells cultured in 3D sphere conditions. Significantly, we found that culture conditions (2D vs. 3D) had a dramatic impact on gene expression programs. Indeed, > 80% of all gene expression variations could be explained by culture conditions whereas MED12 mutations induced gene expression alterations contributed to 12% of variations (Fig. 3h). More importantly, under 3D culture conditions, we observed significantly higher expression of collagen and ECM genes in both WT and mutant cells. However, mutant cells had significantly higher expression (Fig. 3i). Of the 45 collagen genes, 20 of them were expressed at higher levels in 3D conditions, and 15 collagen genes had significantly higher expression in MED12 mutant cells (Fig. 3i) as exemplified in the RNA-Seq tracks for *COL5A3* gene loci (Fig. 3j). Furthermore, global gene expression program under 3D conditions had a higher resemblance to the primary fibroid gene programs. For example, in 2D conditions, we found 166 upregulated and 181 downregulated genes common between MED12 mutant cells vs fibroid tumors. However, these numbers increased to 539 and 569 genes, respectively when cells were cultured in 3D conditions (**Supplementary Fig. 7**). These findings highlight that the impact of MED12 mutations is further enhanced in 3D conditions and the engineered mutations recapitulate induced collagen gene expression, specifically in 3D conditions.

#### The MED12 Gly44 mutation alters DNA synthesis and renders cells sensitive to DNA-damaging agents.

**Figure 4:**
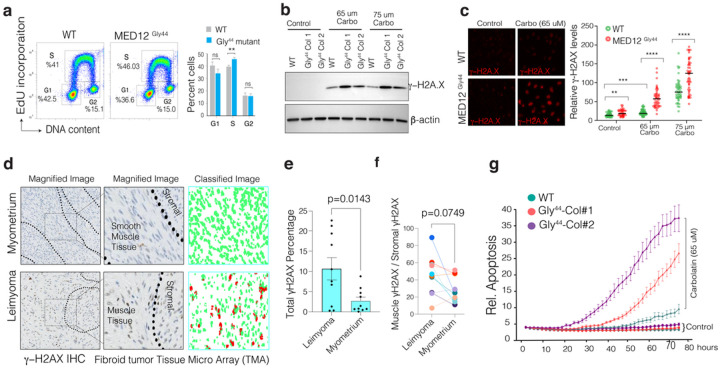
MED12 Gly44 mutations lead to abnormal DNA repair activity and render cells sensitive to DNA-damaging chemotherapeutic agents. **a.** Flow cytometry profiles show the rate of EdU incorporation and DNA content analysis in WT and MED12 mutant cells. **b.** Western blot show g-H2AX and b-actin protein levels in WT and mutant cells. **c.** The images (quantified in dot plots) show relative Immunofluorescence of γ-H2AX signal intensity in control and Carboplatin-treated WT and MED12 mutant cells. **d.** Immunohistochemistry images show γ-H2AX staining in normal and MED12 mutant fibroid tumor tissue microarray. The staining intensity in individual cells was assessed by machine learning-assisted segmentation and quantification (see methods). **e-f.** The dot plots show overall (g) and smooth muscle-specific γ-H2AX IHC signal intensity across ten distinct UF tissue specimens. **g.** Incusyte live-cell imaging results show relative rates of apoptosis (The Incucyte^®^ Caspase-3/7 dye) WT and MED12 mutant cells measured over more than three days in control and Carboplatin-rated cells. (****p<0.0001, ***p<0.001, **p<0.01, Two-sided unpaired t-test)

The above data and cell proliferation rates suggested that MED12 G44 mutation leads to abnormal cell cycle, DNA replication, and repair. We, therefore, tested whether MED12 mutant cells have aberrant cell cycles by analyzing the 5-ethynyl-2′-deoxyuridine (EdU) incorporation and DNA content levels. Notably, the MED12 mutant cells have a significantly higher percentage of cells in the S-phase (p < 0.035), indicating an abnormal rate of DNA synthesis and progression of the DNA replication fork (Fig. 4a, **Supplementary Fig. 8**). These findings support a recent report that MED12 mutant UF samples have increased replication stress due to abnormal progression of the replication fork and increased R-loop formation^34^. Notably, if unresolved, abnormal progression or stalling of replication may lead to structural genomic alterations or excessive DNA damage through replication fork collapse^35,36^. In line with this, the MED12 mutant UF contains the highest number of structural variations in the genome^37,38^; indeed, more than 60% of MED12 mutant UFs have large-scale structural genomic aberrations^39,40^.

The above findings led us to test whether the mutant cells have increased DNA damage at the basal level and are differentially sensitive to DNA-damaging agents such as Carboplatin. To this end, we measured γ-H2AX as a proxy for DNA damage/repair activity. Notably, in line with recent findings^34^, we did not see a significant increase in total γ-H2AX at the basal level by western blot (Fig. 4b). However, the Immunofluorescence staining shows a detectable difference between overall gH2AX signal intensity, indicating a potential DNA damage/repair activity difference between these two cells (Fig. 4c). Interestingly, we saw significantly more DNA damage accumulation in MED12 mutant cells in response to Carboplatin in both MED12 mutant cell lines both by western blot and IF (Fig. 4c, **Supplementary Fig. 9**). The MED12 mutant Fibroid tumors are known to have higher abnormal progression of the replication fork and increased R-loop formation^34^. We, therefore, tested whether primary fibroid tumors also have higher DNA damage/repair activity compared to normal myometrium. We performed an immunohistochemistry analysis of gH2AX on a well-annotated tissue microarray containing normal myometrium and MED12 mutant fibroid tumors (n = 10). To quantify levels if γ-H2AX in a robust and unbiased way, we trained a machine learning algorithm to assess γ-H2AX levels at a single cell level (methods). Critically, we find that MED12 mutant fibroid tumors have detectable and substantially higher overall γ-H2AX levels, indicating higher DNA damage and repair activity compared to normal myometrium (Fig. 4d–e, **Supplementary Fig. 10**). Notably, the γ-H2AX levels within the same fibroid tumors across smooth muscle cells vs. the stromal cells were comparable (Fig. 4f).

The above findings led us to investigate whether the MED12 mutant cells, that display higher activity of DNA repair gene, potentially due to a basal level DNA damage, would be selectively more vulnerable to additional DNA damage. We, therefore, performed long-term live-cell imaging to assess relative apoptosis rates (Caspase 3/7 staining, Biotum) over four days. We observed significant apoptotic cell death selectively in MED12 mutant cells compared to WT cells (Fig. 4g), indicating a potential therapeutically exploitable vulnerability in these mutant cells.

#### The MED12 Gly44 mutations alter genome-wide 3D chromatin organization and genome compartmentalization.

The mediator complex is a critical player in genome organization that links distal regulatory elements to gene promoters^7,8,33,41^. We, therefore, studied whether the abnormal transcriptional program downstream of MED12 mutations is due to altered 3D genome organization. Depending on the scales of organization, the nuclear genome can be categorized into at least three distinct layers in 3D space^42,43^. Globally, the chromosomal DNA is organized into two distinct compartments: A or B. The A compartment is generally associated with gene transcription and active histone modification marks such as H3K27ac and H3K4m3, while the B compartment is mostly composed of heterochromatin. At a finer scale, (usually ~ hundreds of Kb in size), the mammalian genome is organized as topologically associated domains (TAD), whose boundary can prevent the erroneous interactions between the enhancer and wrong target genes. At the finest scale, when a Hi-C library is sequenced deep enough, the chromatin loops that reveals high-resolution promoter-enhancer interactions can be identified. Each layer of genome organization has been linked with proper gene regulation and human diseases^42–45^. To study how MED12 Gly 44 mutation alters the 3D genome organization, we employed high-resolution Hi-C technology and obtained 804,255,654 chromatin contact pairs in WT and 717,644,659 contact pairs in *MED12* mutant cells (**Supplementary Fig. 11a**).

**Figure 5:**
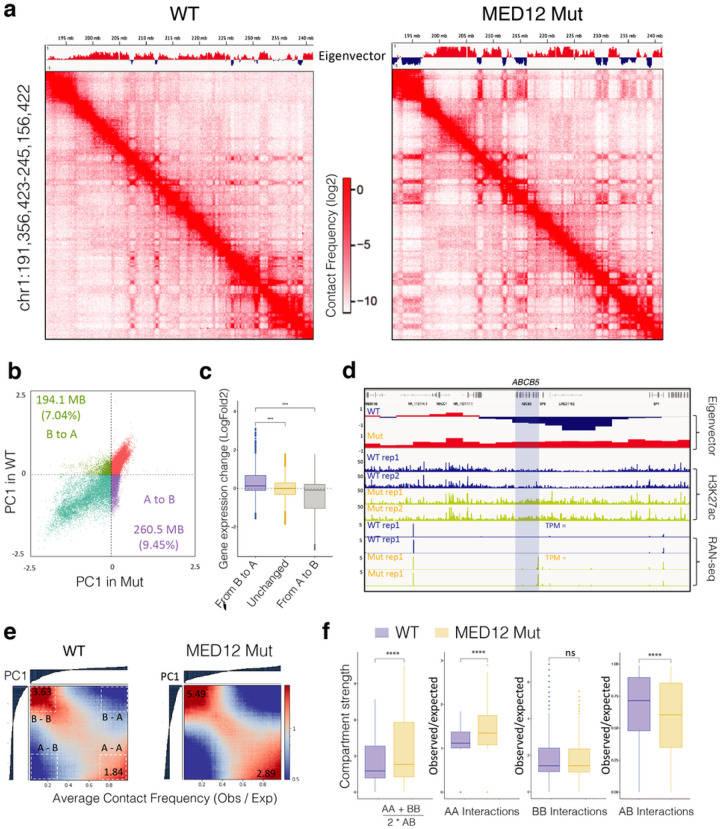
MED12 Gly44 mutations lead to genome-wide A/B compartment reorganization. **a.** Hi-C contact frequency maps at 100kb resolution (chr1: 191M – 245M) in WT (left) and MED12 Mut (right) cells. The first principal component values (PC1) of the genomic regions are shown at the top. **b.** Scatterplot of PC1 in WT versus PC1 in Mut illustrating 194,100,000 bp B to A compartment switch regions and 2605000000 bp A to B compartment switch regions after the MED12 mutation. **c.** The boxplots show the log2 fold change of gene expression (MED12 mutant vs. WT cells) for the genes located within B to A switch, stable, and A to B switch region. The center line represents the median, the box contains the interquartile range, and the whiskers extend to the 5^th^ and 95^th^ percentiles. The statistical test was performed using a two-sided Wilcoxon test (ns, not significant; ***p-value < 0.001, ****p-value < 0.0001).) **d.** A regional example displaying the gene ABCB5 is located in the B to A switch region after the MED12 mutation, along with regional H3K27ac binding enrichment and expression fold change increase. **e.** Saddle plot displays the genome-wide compartment interaction in wild-type (Left) and MED12 Mut (Right) cells based on Hi-C compartment eigenvectors. **f.** The boxplots show the quantification of the compartment strength, A to A, B to B, and A to B interaction strength in WT and Mut cells. The center line represents the median, the box contains the interquartile range, and the whiskers extend to the 5^th^ and the 95^th^ percentiles. The statistical test was performed using a two-sided Wilcoxon test (ns, not significant; ***p-value < 0.001, ****p-value < 0.0001).)

We observed a striking difference on Hi-C maps between WT and Mutant cells. Compared with WT cells, the MED12 mutant cell Hi-C map showed much more pronounced plaided or checkboard patterns (Fig. 5a), suggesting the global change in interactions involved with A/B compartment. We observed significant changes in the compartment state annotations between the two cell types, as exemplified by the eigenvectors track above the Hi-C maps (Fig. 5a). Globally, we found that 7.04% of B compartments switched to A compartment, while 9.45% of B compartments switched to A compartment (Fig. 5b). Integrating gene expression data with 3D genome organization shows that these changes in genomic compartments alters gene expression activity. More specifically, the genes within the compartments that switched from inactive B compartments to active A compartments were significantly upregulated, whereas the genes in the A-to-B switching regions were downregulated (Fig. 5c). For example, the ABCB5 gene, which was in B compartment and silenced in WT cells, was in a B-to-A compartment region and highly expressed in MED12 mutant cells (Fig. 5d)

In addition to the compartmentalization switch, we also observed enhanced 3D chromatin interactions between the compartments of the same types but reduced inter-compartment interactions. As demonstrated in global average contact frequencies across all compartments in the genome (Fig. 5e), MED12 mutant cells have significantly higher intra-compartments (A-to-A or B-to-B) interaction frequencies (Fig. 5f). Interestingly, the global compartment strength is primarily driven by enhanced A-to-A interactions and reduced A-to-B interactions, as B-to-B compartment interactions are comparable in WT and mutant cells (Fig. 5f). Finally, we predicted 23,487 chromatin loops in WT cells and a comparable number of 23,261 loops in MED12 mutant cells, using a machine-learning software that we recently developed^46^. Of these, ~ 20,000 loops were common, and ~ 3000 were cell-type specific chromatin loops (**Supplementary Fig. 11b-c**). These findings indicate that the differential gene expression changes downstream of MED12 mutation are partly due to altered 3D genome organization.

## Discussion

Recurrent somatic MED12 mutations drive fibroid tumors in 70% of cases. Unfortunately, the MED12 mutant cells from these lesions could not be isolated and maintained in culture conditions^47,48^. This formidable challenge limited the ability to create a tractable genetic model system to deeply characterize metabolomics and genomics of pure populations of MED12 mutant cells. In this study, we utilized CRISPR genome engineering to introduce fibroid-relevant recurrent MED12 mutation at the endogenous MED12 locus. We generated multiple pure populations of MED12 mutant cells that recapitulate *in vitro* features of UF fibroid tumors.

Notably, more than 50% of all recurrent MED12 exon two mutations are mutating the Glycine aa at the 44th codon into at least six other aa, indicating that altering Gly at this position results in significant structural and functional alterations in MED12. Interestingly, Gly is the smallest and the only aa that contains Hydrogen as its side chain, whereas all other amino acids contain Carbon^49^. As such, Gly may have a critical impact on both structure and function of the protein because the sidechain-less form of Glycine provides conformational flexibility and can also bind to phosphate, e.g., ATP, in the case of kinase pockets^49^. Our findings also support the clinical^4,50,51^ and mouse genetic^40^ data that the recurrent MED12 Gly 44 mutations are gain of function and “oncogene” in UF tumorigenesis^19^. However, the exact mechanism of how these genetic alterations drive UF pathogenesis is poorly understood.

At the biochemical level, a series of elegant experiments indicated that recurrent MED12 mutations result in altered mediator complex activity. Specifically, through mass spectrometry analysis in insect cells overexpressing UF-linked MED12 mutations, *Turunen et al*. show that MED12 mutations disrupt mediator-associated CDK activity^13^. For example, the same group demonstrated that Gly-44 mutant MED12 disrupts allosteric activation of cyclin C-CDK8/19 complex^12^. Notably, although these findings significantly improved our biochemical understanding of MED12 mutations, how these mutations result in aberrant genomic activity and abnormal expression of thousands of genes is incompletely understood. Our findings in this study shed critical light on the consequences of MED12 mutations in the genome organization and global 3D chromatin interactions.

Supporting previous findings^47,48^, we observe that the MED12 mutant cells have lower fitness in 2D culture conditions; however, in 3D sphere conditions, the mutant cells formed larger spheres and significantly higher expression of ECM and collagen genes, indicating enhanced proliferation and ECM deposition as seen in primary fibroid tumors^1,20,40^. Notably, we identify a potential therapeutically exploitable vulnerability in the mutant cells as these cells are significantly more sensitive to a chemotherapeutic agent, Carboplatin. Our detailed metabolomics and transcriptomics analysis show that the engineered MED12 mutation results in robust metabolic and gene expression changes that are highly reminiscent of primary fibroid tissues. Therefore, the model system we present here may be a valuable asset for the larger research community to study and target UF-relevant MED12 mutations.

Importantly, in addition to being mutated in > 70% of UF^4^, *MED12* exon two mutations are also observed in breast fibroadenomas and phyllodes tumors(59%)^52,53^, uterine leiomyosarcomas (7–30%)^54^, chronic lymphocytic leukemias (5%)^55^, and colorectal cancers (0.5%)^54^. Therefore, whether the mutant MED12 in these diseases also results in similarly altered 3D chromatin compartmentalization is yet to be understood. To this end, the genome-engineered strategy we present here could be explored to introduce similar mutations in other cellular models and comparatively study the outcomes to assess whether the same MED12 mutations result in similar aberrant transcriptomic and 3D genome organization changes.

Our study has some limitations. Firstly, we engineered one of the several MED12 mutations in exon 2. Whether other recurrent hot spot mutations result in the same phenotype is yet to be determined. Secondly, we introduced the MED12 Gly 44 mutation in an immortalized myometrial smooth muscle cell line. Although this model retains specific markers of smooth muscle/myometrium cell markers that are observed in primary cultures of SMC^23^; cell lines are generally a poor model that cannot capture the heterogeneous nature of complex tissues.

## Material And Methods

### Cell lines and culture conditions:

Human myometrial hTERT cells and HUtSMC (ATCC, #PCS-460–011) were maintained in DMEM/F-12 (Gibco, Invitrogen #11320033) with 10% Fetal Bovine Serum (Fisher scientific #SH3091003) and 1 % Penicillin–streptomycin (Life Technologies #15140–122). Cells were cultured and incubated at 37 °C in a humidified atmosphere of 5 % CO2 and 95 % air.

### MED12(G44N) CRISPR knock-in and nucleofection:

*MED12*-G44 targeted sgRNA oligos (CACCGACGGCCTTGAATGTAAAACA/ AAACTGTTTTACATTCAAGGCCGTC) were designed using CRISPOR software^56^ selecting the lenti-guide-puro protocol and were ordered from IDT. 10 μM from each oligonucleotide pair were mixed using annealing buffer (10 mM Tris, pH=8, 50 mM NaCl, 1 mM EDTA) in a total volume of 50 μl and incubated at 95 °C for 5 minutes. Then, they were allowed to slowly cool down to RT. Next, annealed oligos were diluted (1:200) using sterile water. The ligation reaction was performed using BsmB1-digested 50 ng backbone (Modified P2A_mCherry CROPseq-Guide-puro (#86708, Addgene)), 1 μl of the diluted oligos, and 1X T4 DNA ligase and incubated overnight at 16 °C. The next day, 2.5 μl of the ligation reaction was transformed into NEB Stable competent E.coli (C3040H, NEB) and incubated overnight in the presence of ampicillin selection. Next, several colonies were picked and grown overnight. The next day, plasmid DNA was isolated using a Qiaprep spin miniprep kit (27206, Qiagen) and sent to sanger sequencing for validation of successful insertion. Modified GFP-hCas9 (Plasmid #41815, Addgene) and ssDNA-HDR template (IDT) (CTTCCCCCTTCCCCTAAGGAAAAAACAACTAAACGCCGCTTT CCTGCCTCAGGATGAACTGACGGCCTTGAACGTGAAGCAGAACTTC AATAACCAGCCTGCTGTCTCTGGGGATGAGCATGGCAGTGCCAAGAA CGTCAGCTTCAATCCTGCCAAGGTGAGACAACTCTGCCAGGCTGAAGG AAAAGGCTGGAAGAATC) were used as well for nucleofection.

4×10^5 hTERT cells were nucleofected with *MED12*-sgRNA(1.5 μg), Cas9-GFP(1.5 μg) and single strand HDR template(50 pmol) using Neon transfection system (Invitrogen, MPK5000) and Neon 10 μl kit (Thermo, MPK1025). The parameters used for nucleofection were 1400 Voltage/ 20 with/ 2 pulse. 48 hr later, double positive(mcherry+GFP) cells were selected using FACS and seeded as single cells in 96 well plates.

### Single-cell colony qPCR scanning and validation:

Single-cell colonies were split into replicate plates after the colonies grew. One of the replicate plates was washed with PBS twice, Tris-HCL(ph 8.5) was added, and the cells were scraped off with pipette tips. Then, cells were transferred to qPCR plates (#4306737, Applied biosystem). Cells were incubated at 95 °C /15 min for lysis, then cooled on ice for 1 min. Then, they were treated with Proteinase K (55 °C /30 min) (# EO0492, Thermo) and incubated at 95 °C /10 min for proteinase inactivation. After this, they were transferred to new qPCR plates, and the same amount of lysis was used per reaction (reactions were performed as duplicates). qPCR was performed using Fast SYBR^™^ Green Master Mix (4385616, Thermo Fisher) and the same reverse primer (AGGTCATGAAGGCAAACTCAG), with two different forward primers WT/Mut (GCCTTGAATGTAAAACAAGGTTTC/ GCCTTGAACGTGAAGCAGAACTTC) to detect positive colonies.

After detecting mutation-positive colonies, the gDNA was extracted from the replicate plates using the Purelink genomic DNA mini kit (#K182002, Thermo), and RNA was extracted using the Zymo research Quick RNA miniprep kit(#R1054). Isolated RNA was converted to cDNA (#4387406, Applied biosystem). The MED12-exon2 region was amplified using primers F/R (GAAGAGTGATGTTTGAGGGCG/AGGTCATGAAGGCAAACTCAG) from gDNA using primers F/R (CTTCGGGATCTTGAGCTACG/CAGCCAAGTCAGTGAACCAA) from the cDNA, then sent for sanger sequencing for mutation validation.

### CRISPR TIDER analysis:

After sorting for double positive (mCherry+GFP) cells, half of the cells were seeded as a population separately from single-cell colonies. These cells were then used to detect population-level CRISPR knock-in rate using CRISPR-TIDER^57^ analysis. They were subsequently passaged over 9 weeks to determine if MED12 mutation abundance changed in the population over time. Also, mutant positive colonies were analyzed using CRISPR-TIDER to differentiate homozygous/heterozygous mutation. For TIDER, three PCR amplicons were produced following the website’s protocol ( http://shinyapps.datacurators.nl/tider/ ). Control and sample PCR amplicons were produced using F/R (GAAGAGTGATGTTTGAGGGCG/ AGGTCATGAAGGCAAACTCAG) primers on genomic DNA. Reference PCR amplicons were produced using two overlapping primers (ACTGACGGCCTTGAACGTGAAGCAGAACTTCAATAACCAGCC/ AGGCTGGTTATTGAAGTTCTGCTTCACGTTCAAGGCCGTACG) and the same set of F/R primers for the control and sample PCRs described by the website’s protocol. Then, sanger sequencing results (ACGT / NU core) (.ab1 files) were uploaded using the default settings on the website.

### Western Blotting:

Cells were lysed using 1X RIPA buffer, and protein concentrations were determined using the BCA assay (23225, Thermo). 1 μg/ul protein was mixed with 4X sample buffer with reducing agent and boiled at 95 °C for 10 minutes. Next, 20 μg of boiled protein was loaded onto either a NuPAGE 4–12%, Bis-Tris gradient gel (#NP0335, Thermo) or 3–8%, Tris-acetate gel (#EA0375, Thermo), and samples were run at 130 V for about 1.5 hours. Proteins were transferred to nitrocellulose membrane using iBlot dry transfer system (Program 3 / 8 min). Next, membranes were blocked using 5% milk dissolved in TBS-T (20 mM Tris, 150 mM NaCl, 0.1% Tween 20; pH 7.6) for 1 hour, rocking at room temperature (RT). After blocking, membranes were incubated with primary antibodies (1:1000 dilution) (MED12(Bethyl lab, #A300–774A), TDO2 (Protein-tech,#15880–1AP), Phospho-Histone H2A.X (Ser139)(Cell Signaling,#2577), Anti-β-Actin antibody (Sigma, Mouse monoclonal, #A1978–100UL)) prepared in blocking buffer overnight at 4 °C. The next day, membranes were washed with TBS-T 3 times for 5 minutes. Then, they were incubated with secondary antibodies (1:10000) (Anti-Rabbit IgG (H+L) (Promega,# W4011), Anti-Mouse IgG (H+L)(Promega, #W402B)) diluted in blocking buffer for 1 hour at RT. After the incubation, membranes were again washed 3 times for 10 minutes. Lastly, membranes were covered with western blot detection reagents (37074, Thermo Fisher) and visualized using the iBright imaging system.

### RNA seq and Differential expression/GSEA analysis:

Total RNA was isolated using the Zymo research Quick RNA miniprep kit utilizing the on-column DNAse treatment according to the manufacturer’s instructions (#R1054). RNA was prepared for sequencing using the NEBNext^®^ Poly(A) mRNA Magnetic Isolation Module (NEBNext #E7490) and NEBNext^®^ Ultra^™^ Directional RNA Library Prep Kit (NEBNext #E7420) according to the manufacturer’s instructions. Paired-end sequencing of all RNA libraries was performed on the Illumina NextSeq 500 Platform.

The quality of FastQ files of RNA seq was checked using FastQC (www.bioinformatics.babraham.ac.uk). RNA-seq reads were aligned to the GRCh38 human genome assembly (Ensembl release 102) using the STAR aligner (v2.7.5)^58^ with default settings. BAM files were converted into bigwig files using bam coverage/DeepTools(v3.5.1)^59^ (bin size 1, normalized BPM). Gene exon counts were found using featureCounts (Subread package, v1.6.1, settings: -g gene_id -t exon -p -s 2)^60^. Differential expression analysis was performed using the R package DESeq2 (v1.36.0)^61^, and the Wald test was used for significance (P value). The IHW method was applied for multiple testing corrections, with the false discovery rate controlled for at (<0.05). Differentially expressed genes in the MED12(G44N) cells (Padj<0.01) and DE genes in Leiomyoma cells (Padj<0.01) were used for GO term analysis which was performed using the NIH’s DAVID Bioinformatics Resource^62^(**Supplementary Figure 6**). DESeq2 normalized exon counts were used in GSEA (v4.0.2)^63^ analysis in default settings.

All leiomyoma and myometrium RNA-seq data were downloaded from the NCBI-GEO data repository via accession GSE128242 and original publication data processing steps were followed^33^. Heatmaps of differentially expressed genes were plotted in R (cran.r-project.org) using pheatmaps (Kolde, Raivo / v.1.0.12, 2019).

### Incucyte live cell imaging:

Incucyte Live cell imaging system (Sartorius) was used for tracking cell proliferation. The system took a photo of cell plates every two hours in different image channels (Phase/Green or Red). For cell nucleus counting, 1 μM SiR-DNA nuclear dye was used (Cytoskeleton, #SC007) and captured using the red channel. At the end of the experiment, proliferation data were analyzed using the Incucyte analysis tool and p-values were calculated using the incucyte raw data. Relative proliferation was normalized to the starting time.

### EdU staining:

Cells (4×10^5) were seeded in six-well plates one day before Edu staining. Then, cells were treated with 10 uM Edu for 90 minutes following the manufacturer’s protocol (Click-iT^™^ Plus EdU Alexa Fluor^™^ 488 Flow Cytometry Assay Kit, Thermo,# C10632). Next, cells were stained for DNA content using FxCycle^™^ Violet Stain (Thermo, # F10347)(1 μl violet for 1 ml media). Lastly, cells were analyzed using flow cytometry, and the results were analyzed using FlowJo.

### H3K27Ac cut & tag and analysis:

Benchtop CUT&Tag^31^ V.2 protocol was slightly Modified to pro le genome-wide H3K27ac. Brie y, Concanavalin A-coated (ConA) beads (10 μl/sample) were washed using binding buffer (100 μl/sample) twice and kept on ice until the cells were ready. Cells were then harvested and counted to obtain 100,000 cells for each sample. Then, cells were centrifuged and washed one-time using wash buffer. After washing, cells were centrifuged and resuspended in wash buffer (100 μl/sample). Next, ConA beads were added to each sample while vortexing gently. The bead-sample mixture was rotated for 10 minutes at RT. Next, samples were put on a magnet stand to clear the liquid. Following this, samples were resuspended in ice-cold antibody buffer (50 μl/reaction) while vortexing and then kept on ice. Afterward, 3 μl H3K27ac antibody (ab4729, Abcam) was added to each sample while vortexing gently. Samples were incubated overnight at 4 °C on a nutator. The next day, samples were cleared using a magnet stand. Secondary antibody (ABIN101961) mixture (2 μl antibody diluted in 100 μl dig-wash buffer for each sample) was added to each sample while vortexing. After 1 hour of incubation at RT on a nutator, samples were cleared and washed twice using Dig-wash buffer (1 ml/sample). Then, the pA-Tn5 adapter complex (2.5 μl pA-Tn5 in 47.5 μl Dig-300 buffer) was added to each sample while vortexing and samples were incubated for 1.5 hours at RT on a nutator. Here, we used pAG-Tn5 from EpiCypher (Cat No: 15–1117). After the incubation, samples were cleared and washed twice using Dig-300 buffer (1 ml/sample). Next, 300 μl tagmentatiton buffer was added to each sample, and they were incubated for 1.5 hours at 37 °C. To stop tagmentatiton, 10 μl 0.5M EDTA, 3 μl 10% SDS, and 2.5 μl 20 mg/ml Proteinase K was added to each sample. After adding Proteinase K, samples were vortexed immediately and incubated overnight at 37 °C. The following day, samples were incubated at 50 °C for 30 minutes. To isolate DNA, 300 μl phenol-chloroform was added to samples and mixed by vortexing. Each mixture was transferred into a phase-lock tube (129046, Qiagen) and centrifuged at 16,000 g for 3 minutes at RT. Next, 300 μl chloroform was added to each sample and inverted 10 times to mix. Samples were then centrifuged at 16,000 g for 3 minutes at RT. After centrifugation, the aqueous layer was transferred to new tubes containing 750 μl 100% ethanol and mixed well with pipetting. Samples were incubated on ice for 5 minutes and centrifuged for 15 minutes at 4 °C 16,000 g. The liquid was removed (pellet may not be visible), and 1 mL 100% ethanol was added to rinse the pellet. Then, samples were centrifuged for 1 minute at 4 °C 16,000 g. The liquid was carefully removed, and samples were air-dried for about 15 minutes. Next, the pellet was dissolved using 25 μl TE buffer (10 mM Tris-HCl pH 8, 1 mM EDTA supplemented with 1:400 diluted RNAse A). To remove potential RNA contaminants, samples were then incubated 10 minutes at 37 °C. Library preparation was performed as described in Benchtop CUT&Tag V.2 protocol. The library was sequenced using NextSeq 500 (2 × 75 bp) to obtain 5 million reads per sample.

Reads were aligned to the hg38 reference genome using Bowtie2^64^. Then, PCR duplicates and blacklisted regions were removed using Picard tools (“Picard Toolkit.” 2019. Broad Institute, GitHub Repository. https://broadinstitute.github.io/picard/) and bedtools, respectively. Bigwig files were generated using deepTools^65^ to visualize CUT&Tag tracks. Peak calling was performed using MACS2. To determine differentially acetylated regions, we used DiffBind (Stark R and Brown G (2011)/Bioconductor)^66^ package available on R studio.

### Apoptosis assay:

Cells were seeded into 96 well plates at a density of 1.5 × 10^3^ cells/well. The following day, treatments were performed using 65 uM/75um Carboplatin (IC30/IC40 concentration, (Selleckchem,# S1215) mixed with 1:1000 diluted Caspase 3/7 dye (10403, Biotum). Then, cells were monitored using the Incucyte live cell imaging system using phase and green channels. The apoptosis rate was determined using the green integrated intensity/confluency values, and the results were plotted using the Incucyte cell imaging analysis.

### Immunofluorescence staining:

Approximately 1.5×10^3^ cells were seeded onto coverslips in 6-well plates. The next day, cells were treated with the indicated concentrations of Carboplatin for three days. Then, cells were washed with PBS and fixed using 4% paraformaldehyde in PBS for 10 minutes at RT. After fixation, cells were washed three times with ice-cold PBS and incubated with 0.25% Triton X-100 in PBS for permeabilization. Next, cells were washed three times for 5 minutes with PBS and blocked using 1% BSA, 22.52 g/mL glycine in PBS-T (PBS+0.1 Tween 20) for 1 hour at RT. After blocking, cells were incubated with primary antibodies (Phospho-Histone H2A.X (Ser139)(1:1000)(Cell Signaling,#2577)) prepared with 1% BSA in PBS-T overnight at 4 °C in a humidified chamber. The next day, cells were washed three times for 5 minutes with PBS-T, and then they were incubated with a secondary antibody (Alexa Fluor 594, Invitrogen #A-11012) prepared in 1% BSA in PBS-T for 1 hour at RT. Afterward, cells were washed three times for 5 minutes using PBS-T. Next, coverslips were mounted onto microscopy slides using a mounting medium with DAPI (S36939, Thermo Fisher). Finally, slides were visualized using the EVOS cell imaging system, and the images were analyzed using ImageJ software. Relative γH2AX levels were drawn and the p-value (Two-sided unpaired t-test) was calculated in Prism-GraphPad (9.4.1).

### Liquid chromatography-mass spectrometry (LC-MS) and analysis:

Cells were seeded on 10 cm plates and the medium was completely aspirated once they reached ~70–80 % con uence. Then, cells were rinsed with ice-cold PBS twice and add 1 ml 80% (vol/vol) methanol (cooled - 80 °C). Then, cells were scraped on ice with a cell scraper and collected lysate in a conical tube. The lysate was incubated at −80 °C for 5 min and vortexed at room temperature for 1 min. Repeat that step two times. Then, the lysate was incubated at −80 °C overnight for protein precipitation. The next day, they were centrifuged at 20000xg for 15 mins at 4 °C. Transfer supernatant which contained metabolites to a new 1.5 ml conical tube. Then, the pellet was fully dissolved in 8M urea buffer, and protein concentrations were determined using a BCA assay (Thermo, #23225). The same amount of metabolomes based on protein amount were submitted for LC-MS. The metabolome was analyzed in the NU metabolomics core facility. Then, all metabolome peak areas of samples were normalized to their TIC (Total ion count). And, normalized peak area per metabolite results were analyzed on Metaboanalyst 5.0^67^ (https://www.metaboanalyst.ca/).

### High-throughput chromosome conformation capture (Hi-C) and data processing:

The Hi-C was performed using the Arima-HiC Kit (A510008, Arima Genomics) as instructed by the manufacturer. Approximate 1 million WT/Mut cells were harvested, counted, and fixed with 1% formaldehyde and quenched with 0.125 M glycine at room temperature. The fixed cells were digested with the restriction enzyme and end-labelled with Biotin-14-dATP, followed by proximity ligation. Then reverse-crosslinking was performed to the ligated samples and sheared into 300–500bp fragments. The Biotin-labeled DNA fragments were then end-repaired following adapter ligation and PCR amplification. Hi-C libraries were generated using KAPA Library Quantification Kit (KAPA Biosystems) and quality-checked according to the manufacturer’s protocol.

The mapping, filtering, and binning of the data were done using the runHiC(v0.8.6) pipeline. First, the adapters of the Hi-C FASTQ files were trimmed using Trim Galore(v0.4.5), and then runHiC aligned the trimmed FASTQ files to the hg38 human reference genome with Burrows-Wheeler Aligner. Then, low-quality reads and PCR duplicates were removed. Read pairs were then used to couple aligned reads, and redundant PCR artifacts and read pairs aligned to the same restriction fragments were filtered out before the next stage. The binning stage binned the reads at 5-kb, 10-kb, 50-kb, 1-Mb, 10-Mb, and 50-Mb resolution and performed the ICE normalization at the same time. After the binning state, ICE normalized matrices .mcool files were generated for downstream analyses.

### High-throughput chromosome conformation capture (Hi-C) Compartment Analysis:

The compartment analysis for WT/Mut HiC was performed using cooltools. The A/B compartments PC1 values at 100-kb resolution were called using cooltools eigs-cis command. The scatter plot of PC1 values before and after the MED12 Gly44 mutation was plotted using the ggplot2 package of R (v4.1.3). The regions with positive PC1 values were identified as compartments A. The regions with negative PC1 values were identified as compartments B. The compartment strength, AA interactions, BB interactions and AB interactions were calculated and visualized using boxplots with Wilcoxon signed rank test for statistical analysis. The compartment strength was visualized using the saddle plot function implemented in cooltools.

### 3D Spheroid formation and co-culture experiment:

Cells were seeded (3000 cells per well) on Corning ultra-low attachment plate, 96 wells (#4515), and spheroid photos were taken using the EVOS (ThermoFisher Cat# AMF5000) cell imaging system. Then, spheroid volumes were calculated using ImageJ. Results were plotted using Prism software. For the co-culture spheroid experiment, WT, MED12(G44N) mutant, and MED12(KO) hTERT cells were transduced with lentivirus-included mCherry plasmid. For viral production, HEK293T cells were seeded into a 10 cm plate 1 day before transfection. 1 ug pMD2.G (Addgene, Plasmid #12259), 2 ug psPAX2 (Addgene, Plasmid #12260) and 4 ug of the Modified P2A_mCherry CROPseq-Guide-puro (#86708, Addgene) plasmid were co-transfected into HEK293T cells using PEI. Media was refreshed 12 h after transfection. The virus was collected 24 and 48 h after the first media refreshment and filtered through a 0.45 mm filter. For viral transduction, cells were incubated with virus solution diluted in media and supplemented with 0.01mg/mL polybrene for 24 h. After transduction, the mCherry-positive cells were sorted by flow cytometry.

For the co-culture spheroid experiment, the same number of HUtSMC (ATCC, #PCS-460–011) and mCherry positive hTERT cells (WT / Mut / KO) were seeded together in Corning ultra-low attachment plates (1500/1500 cells per well). After four days, spheroid photos were taken using the EVOS cell imaging system and spheroid volumes were calculated using ImageJ. Then, spheroids were collected and the cells were dispersed using trypsin-EDTA (0.25%) (Gibco,# 25200056). Then, the mCherry positive cell rate in the population was calculated using ow cytometry.

### High-resolution analysis of Tissue microarrays:

The comparison of γH2AX staining ratios between the tissue microarray samples was carried out using Machine Learning. The samples were cropped with identical settings using QuPath Software^68^. The images were then segmented through the Trainable WEKA Segmentation^69^ plug-in of Fiji Software^70^. The γH2AX (Phospho-Histone H2A.X (Ser139) (20E3) Rabbit mAb (Cell signaling, #9718)) stained and γH2AX non-stained nucleus shapes were aligned to the plug-in. Thus, classified images were generated following the alignments. The colored pixel distribution of each classified image was obtained through the Image Color Summarizer and the pixel counts of green shapes (indicating unstained nuclei) and red shapes (indicating γH2AX stained nuclei) were compared.

To understand the γH2AX stained nuclei distribution between stromal and myometrial tissues within each sample, a semi-automated analysis was used. Following manual separation of the stromal and myometrial cell types, the nuclei were highlighted on different gradients according to their staining, through Photoshop’s Magic Wand tool. The highlighted particles then were evaluated using Fiji software, giving the unstained nucleus counts and γH2AX stained nucleus counts on different tissues of each sample.

### H3K27Ac cut-tag analysis:

Differential binding analysis was performed using the R package Diffbind (Stark R and Brown G (2011)/Bioconductor)^66^. Enriched and depleted peaks were chosen using a threshold of <0.1 FDR. Heatmaps were generated using Deeptools’^65^ computeMatrix and plotHeatmap functions. Peaks were annotated using the R package ChipSeeker^71 72^. Genes were determined to be peak-adjacent if the TSS or TES were within 10 kb of the peak center. RNA-counts were normalized with the R package DESeq2^61^ using variance stabilizing transformation (VST). Violin plots of normalized RNA expression were generated using the R package ggplot2 (H. Wickham/ggplot2/Springer/2016) and p-values were calculated using Wilcoxon signed-rank tests.

## Data Availability

All metabolomics and gene expression data will be made publicly available.
